# The past, present and future of genomics and bioinformatics: A survey of Brazilian scientists

**DOI:** 10.1590/1678-4685-GMB-2021-0354

**Published:** 2022-06-01

**Authors:** Mariana Rocha, Luisa Massarani, Sandro José de Souza, Ana Tereza R. de Vasconcelos

**Affiliations:** 1 Technological University Dublin, School of Computer Science, Dublin, Ireland.; 2 Fundação Oswaldo Cruz, Instituto Nacional de Comunicação Pública da Ciência e Tecnologia, Rio de Janeiro, RJ, Brazil.; 3 Universidade Federal do Rio Grande do Norte, Instituto do Cérebro, Natal, RN, Brazil.; 4 Universidade Federal do Rio Grande do Norte, Centro Multiusuário de Bioinformática, Natal, RN, Brazil.; 5 Sichuan University, Institutes of Systems Genetics, West China Hospital, Chengdu, China.; 6 Laboratório Nacional de Computação Científica, Laboratório de Bioinformática, Petrópolis, RJ, Brazil.

**Keywords:** Genomics, bioinformatics, Brazil, natural language processing, researchers’ perceptions

## Abstract

Brazil has one of the highest rates of scientific production, occupying the ninth position among countries with genome-sequencing projects. Considering the rapid development of this research area and the diversity of professionals involved, the present study aims to understand the expectations, past experiences and the current scenario of Brazilian research in bioinformatics and genomics. The present research was carried out by analyzing the perceptions of 576 researchers in genomics and bioinformatics in Brazil through content and sentiment analysis techniques. This group of participants is equivalent to 48% of the members of the research community. The results suggest that most researchers have a positive perception of the potential of this research area. However, there is concern about the lack of funding for investing in equipment and professional training. As part of a wish list for the future, researchers highlighted the need for higher funding, formal education, and collaboration among research networks. When asked about genomics and bioinformatics in other countries, the participants recognize that sequencing technologies and infrastructure are more accessible, allowing better data volume expansion.

## Introduction

The year 2021 marked the 20^th^ anniversary of the publication of two drafts of the human genome sequence: one by the Human Genome Project (Lander et al., 2001) and the other by a company, Celera Genomics (Venter et al., 2001). From discovering the DNA to sequencing all 3 billion letters of a human genome, research in genomics and bioinformatics has provided insights into various biological features, such as cellular metabolism, molecular biology, species evolution, and pathology (Weissenbach, 2016). Furthermore, sequencing the genome is relevant not only for biologists but also for different actors in science. For example, the advent of next-generation sequencing (NGS), a direct consequence of the first two decades of genomics, led to an exponential influx of data and increased the computational challenge associated with data processing and analysis (Gauthier et al., 2019). In addition, developing countries increased the scientific productivity in the area, providing more ethnic diversity in genome studies, a relevant need mainly when genomics technologies are used to identify rare diseases or predict human responses to new drugs (Bustamante et al., 2011). 

In spite of the fact that a bias still exists, genomics studies focusing on specific populations have been advancing in the last few years. In the first years of the genomics era, most geneticists directed their research to analyze European populations and their needs. Nowadays, the growth of genomics studies over racial and ethnics minorities provided the possibility of spreading the benefits of the area, including initiatives such as the Special Programme for Research and Training in Tropical Diseases (TDR), a program for scientific collaboration that support efforts to combat tropical diseases (Morel, 2000). Take, for example, the research environment for bioinformatics and genomics research in Latin America. This region, composed of 20 countries and 14 dependent territories, had a relevant growth in scientific productivity, showing a continuous increase from 2000 (De Las Rivas et al., 2019). This trend is not particular to Latin American countries, as studies around life science technologies started to grow world-wide during the early 2000s (De Las Rivas *et al*., 2019). However, Latin American countries’ scientific production in genomics was particularly benefited by the establishment of a number of research networks. In 2009, the Iberoamerican Society for Bioinformatics (SoIBIO) was launched (De Las Rivas *et al.*, 2019) and, since 2010, the International Society for Computational Biology (ISCB) began organizing the ISCB Latin America Conference on Bioinformatics. Both initiatives are intended to contribute to the field’s growth within this region and support scientific innovation across Latin America. However, the fast development of research in the area caught Latin American researchers unprepared a decade ago. The need for sophisticated technology and large-scale database management made it challenging to deliver results at the same level as European and north-American countries countries, resulting in difficulties in communicating those on good or regular impact journals (Ramírez et al., 2002). Recent studies suggest the challenges have not changed. For example, recent research conducted in Mexico revealed that experts in Bioinformatics believe the lack of technological infrastructure and human resources are deficiencies in the research area (Armenta-Medina et al., 2020). Another study carried out by consulting experts in the field revealed that researchers from different regions of Brazil deal with unequal access to sequencing platforms, especially for the country’s northern area (Bicudo, 2016). Due to these difficulties, Latin American countries would need to double their research effort to approach the average world scientific production in the field (De Las Rivas *et al.*, 2019).

In Latin America, Brazil has a high rate of scientific productivity in genomics and bioinformatics. In an analysis of scientific papers on bioinformatics published by Latin American authors between 1991 and 2016, De Las Rivas et al. (2019) identified 2119 publications - more than half of those (1068) were published by Brazilian authors. The turning point in the history of genomics in Brazil was the complete sequencing of the citrus pathogen *Xylella fastidiosa* in 2000 (Simpson et al., 2000). For the first time, a phytopathogen was sequenced, revealing pathogenicity mechanisms. This discovery supported developing solutions to control the damage caused on oranges, grapevines, citrus and coffee commercial production. Nevertheless, this result stimulated the development of new sequencing projects in the country (Simpson, 2001). A relevant aspect that is usually related to the success of this research field in Brazil was the setup of research networks (Xavier et al., 2008). Some examples are the Organization for Nucleotide Sequencing and Analysis (ONSA), launched by the São Paulo Research Foundation (FAPESP) in 1997 (Simpson and Perez, 1998), and the Brazilian National Genome Network, launched by the Brazilian National Council for Scientific and Technological Development (CNPq) in 2000 (Simpson *et al.*, 2004), first to be undertaken on a national scale. Many other regional initiatives have been launched in the past two decades, which allowed the country to have an installed capacity not only for sequencing but also for the analysis and interpretation of data. Recently, Brazilian researchers have been quick to characterize the first sample of the SARS-CoV-2 virus, which caused the COVID-19 pandemic (Araujo et al., 2020; Jesus *et al.*, 2020), and to study the spread of the virus in the country since March 2020 (Candido et al., 2020; Silva Francisco Jr et al., 2020; Buss et al., 2021; Faria et al., 2021; Voloch et al., 2021). According to (Chasapi et al., 2020), Brazil is part of the ranking listing of 40 leading countries in bioinformatics, and occupies position 23 of the top 1% of highly cited papers. Although research in bioinformatics and genomics in Brazil had positive and relevant features during the last two decades, the country still faces challenges common to developing countries, such as the lack of funding, depending on researchers’ creative approaches to achieve their research goals (Patrinos et al., 2020). This study aims to understand the expectations, past experiences, and the current scenario of Brazilian research in bioinformatics and genomics by gathering researchers’ perceptions about the field.

## Methods

### Research design

The present study investigates the experts’ perceptions of the past, present, and future of genomics and bioinformatics in Brazil. The approach adopted to achieve this goal was to gather and evaluate the perceptions of Brazilian professionals working in the field. Perception can be defined as how “we see things”, and it influences people’s opinion and understanding about a situation (Given and Saumure, 2008). Our hypotheses were based on the fact that understanding expert perceptions about their field, especially when considering a diverse group of professionals, can provide valuable information about the challenges, achievements, and relevance of that research area. 

The current study was developed to address the following research question (RQ): What do Brazilian professionals working in the field think about the past, the present, and the future of genomics and bioinformatics? To better structure our work and considering this research’s multidimensional character, the main RQ was divided into three sub-questions to better structure our work and consider this research’s multidimensional character. Table 1 describes the sub-questions, objectives, and methods to be adopted to answer each of those. The methodology is described in detail in the data analysis section.

**Table 1-:** Sub-questions and research objectives.

Sub-questions (SQ)	Objectives
SQ1: What is the profile of the researchers currently working in the field?	To collect and evaluate details about genomics and bioinformatics professionals considering demographic features, educational background, and professional experience
SQ 2: What are the perceptions about the present and future? How does it confront the perceptions about the area in other countries?	To collect and evaluate genomics and bioinformatics professionals’ thoughts on the current and future situation of the field both in Brazil and abroad.
SQ 3: What are the milestones achieved in the area, and what are the expert wishes for the future?	To collect and evaluate information about the country’s main achievements and what should be considered for future work.

### Data collection

The data was collected through an online questionnaire (Appendix) containing 25 questions, 19 closed-ended and six open-ended. The first closed-ended question asks how long the respondent is working in the area of genomics and bioinformatics. If the participant selected the option that states s/he is not working in the area, the questionnaire was then ended. This procedure allowed the exclusion of researchers that are not from the aforementioned area. Participants were recruited by convenience sampling, being contacted by email or phone. The questionnaire was shared with postgraduate courses in the area, short courses and specializations, national networks of professionals, research groups, and contacts in the industry. The questionnaire was available from February to August 2020. The data could be provided anonymously, but researchers had the option of providing their names and email for future contact. 

### Participants’ profiles

To answer the SQ1, the first phase of data analysis focused on gathering an overview of participants’ profiles through a descriptive analysis of the data. This analysis considered only the data generated by the answers provided on the close-ended questions. Aspects such as participants’ gender, the state of Brazil where they live, number of years working in the area of genomics and/or bioinformatics, and other details were included. 

### Participants’ perceptions

The data generated by the answers to the open-ended questions were evaluated by two main methods to answer SQ2. First, semantic and lexical analysis of the answers provided by the participants was conducted using the QDA Miner software, developed by Provalis Research of Montreal, Canada. QDA Miner is a full-featured software package for coding, searching, and analyzing mixed-model data (Lewis and Maas, 2007). Using this software, we attempted to identify the words most commonly used by the researchers, considering their frequency on the participant’s answers. This was done after “stemming the words”, a linguistics procedure to reduce all words with the same stem to a common form (Lovins, 1968). Also, common words, also known as stop words, were excluded from the pool. This process allowed us to focus on the meaningful words, removing very common ones such as “a”, “with”, “at” and “on”. We used a built-in list provided by WordStat, but also added extra words, such as “bioinformatics” and “genomics”, which appeared many times as this was the focus of the survey.

Besides retrieving the frequency of the words, we also used QDA Miner to evaluate the groups of words that tend to show up together in the same sentence, forming clusters. Data clustering is performed based on Jaccard’s coefficient, a statistics measurement to identify similarities between texts (Niwattanakul et al., 2013). We also used the WordStat text mining package from Provalis Research to perform a content analysis of the answers to two questions of the questionnaire. Content analysis is a systematic, objective, quantitative analysis of text characteristics (Neuendorf and Kumar, 2015). The first question aimed to collect what the respondents believed to be milestones of the development of genomics and bioinformatics in Brazil. In the second question, each participant was asked to create a wish list containing their expectations for the future in the field. The responses were carefully read so patterns could be identified and codes created. After that, the responses were classified according to the codebook created. The codes were used to classify each sentence of a response. The same answer could contain a number of codes, and the same code could appear twice in the same response (case) if identified in different sentences.

Sentiment analysis was the second main method applied to evaluate the open-ended questions. Sentiment analysis is a technique that classifies sentiments/opinions identified in texts. These sentiments are usually classified as positive, negative, or neutral. This process helps determine how a specific population perceives a context, a product, public policies, and other social aspects (Prabowo and Thelwall, 2009). We adopted the lexical-based sentiment analysis, where the data collected was classified according to a predefined list of words, where each word is associated with a specific sentiment (Gonçalves et al., 2013). The analysis was made based on the OpLexicon (Souza et al., 2011), a Portuguese language lexicon constituted around 15,000 words classified by their morphological category and with polarities positive, negative, and neutral. Unlike other dictionaries, OpLexicon is composed not only of adjectives but also of different types of words, providing better accuracy (Souza and Vieira, 2012). Before classifying the data, a number of procedures were performed. The pre-processing phase included the use of Python libraries to access, clean, and manipulate the dataset. The dataset was stored on Google Sheet and gspread (https://docs.gspread.org/en/v4.0.1/) library was used to access it. The data analysis library pandas was used to transform the dataset into a data frame, avoiding missing values. The dataset was then converted into lowercase text using the method *lower()*, and the Python module string (https://pandas.pydata.org) was used to clean the dataset by identifying and removing punctuations. The module re (https://docs.python.org/3/library/re.html) performed a tokenization process, separating each response of the dataset into individual words. To simplify the text, the package Natural Language Toolkit (NLTK, https://www.nltk.org) was used to remove stop words, such as “a”, “with”, “at”, and “on”. As the dataset was ready for analysis, urllib (https://docs.python.org/3/library/urllib.html) package was used for accessing the file containing the OpLexicon dictionary and io (https://docs.python.org/3/library/io.html) module was used to prepare this file for the analysis. Finally, matplotlib (https://matplotlib.org) was adopted for the visualization of the final results. This pre-phase process is relevant considering how the classification using OpLexicon works. Each word (token) of the text is classified according to the OpLexicon dictionary - positive words receive a score of 1, negative words receive a score of -1, and neutral words receive a score of 0. After that, all the scores of the words in the response are summed up and, if the resulting value is higher than 0, the response is positive; if lower than 0, the response is negative; if equals to 0, the response is neutral. Both the pre-process and sentiment analysis phases were carried out and are available on Google Colab (https://colab.research.google.com/drive/19UF9PhYCgd6JvryVjiC_vZWe5K8DlD1a), a free, cloud-hosted Jupyter notebook that allows developers to write, execute, and share Python code.

## Results

A total of 576 participants answered the questionnaire. To estimate the approximate number of Bioinformaticians in Brazil, we cross-referenced the ones who answered this survey, those who are or were part of the Brazilian Association of Bioinformatics and Computational Biology (AB3C) in the last 10 years, and the number of masters and doctoral students that finished or were/are enrolled in graduate courses (800 students). It is worth noting that we removed the duplicity to have a number closer to reality. Considering these criteria, we estimated that the area includes around 1200 researchers and students in Brazil, and our pool of respondents represents approximately 48% of the population active in the field. After collecting the data, the first step consisted of a data cleaning process to remove empty and duplicates. This resulted in a collection of 541 responses. 

### Participants’ profiles

**Figure 1-:**
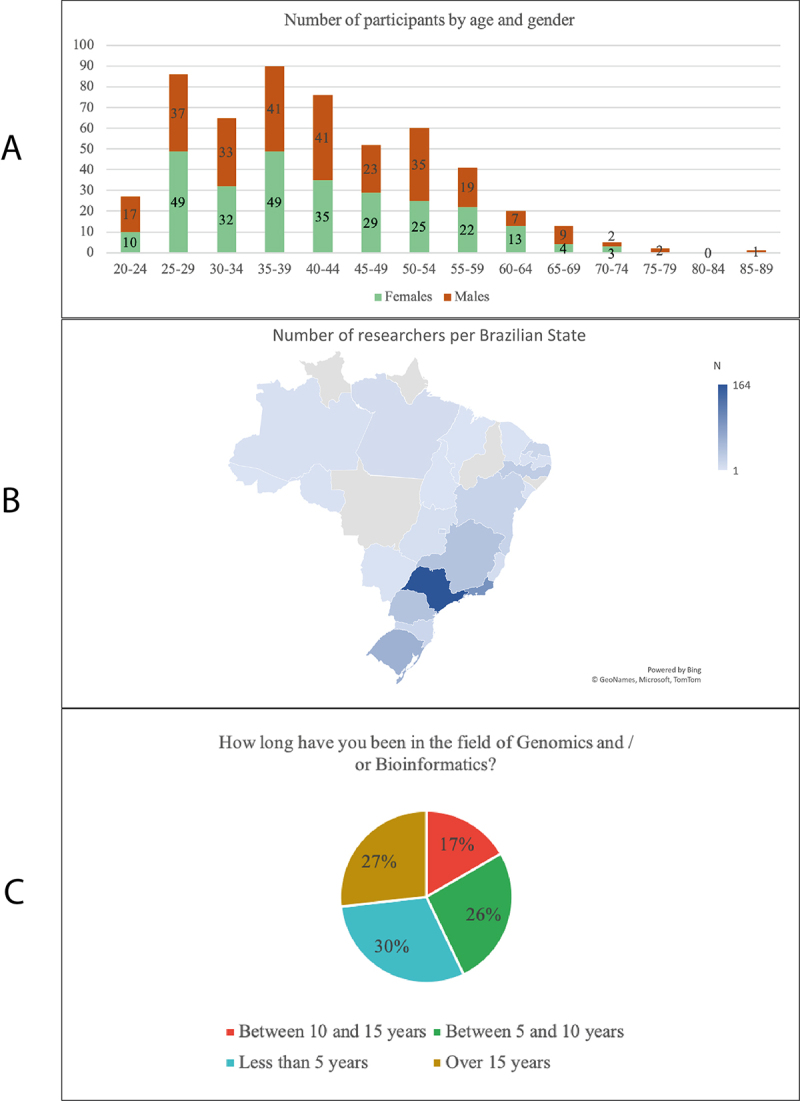
Profile of study’s participants. (A) The number of participants according to gender and age range. (B) Distribution of the participants according to the Brazilian state where they live. (C) Percentage of participants according to their time working in the field of genomics and/or bioinformatics.

From the collected answers, 50.1% were provided by female researchers, 49.5% by male researchers, and 0.4% of participants preferred not to declare their gender. Most of the participants were between 35-39 years old (90 participants, 16.6%), followed by participants between 25-29 years old (86 participants, 16.1%). These data are shown in Figure 1A. 

Most of the participants currently live in Brazil (430 researchers, 97.5%), with most of them concentrating in the São Paulo state (38.0%). The results also show that 11 participants live in other countries, such as the United States, Argentina, and China. Figure 1B illustrates the distribution of the participants around Brazil. When answering how long they are working in the field, 30.0% of participants said to be working in the area for less than 5 years, while 27% of participants do research in Genomics and/or Bioinformatics for over 15 years (Figure 1C). Most of the respondents (94.0%) believe their research is extremely relevant to the area.

Participants were asked about their previous/current education, considering aspects such as if they attended private or public institutions and how long they have been graduated. When referring to their high school education, 47.0% of the respondents said to have attended a public institution, 41.0% attended a private institution, and 11.0% had part of their high school studies in public and part in a private institution. When talking about their university studies, 89.0% attended public institutions, 7.0% had part of their studies in public and part in a private institution, and 4.0% attended private university institutions. Considering the time since they have finished their studies, most of the participants finished their courses over 10 years ago (Table 2).

**Table 2 -:** Answer to the question: “Mark in the table below, for all levels of education, if you are attending or have already completed and in case you have already completed what was the year of completion”.

Time since completion	Bachelor’s degree	Specialisation	Master’s degree	PhD	Post-doctorate
Over than 10 years ago	64.5%	18.9%	44.9%	41.0%	22.6%
Between 05 and 10 years	15.5%	2.8%	13.3%	14.6%	12.2%
Up to 05 years	17.2%	3.5%	19.2%	14.4%	12.8%
Over 3 years ago	0.2%	0.0%	0.0%	0.2%	0.0%
Studying	0.9%	1.1%	7.8%	16.1%	10.2%

The most popular areas of formal education among researchers in bioinformatics and genomics is biological science (35.0%), followed by biotechnology (13.0%) and biodiversity (7.0%). The majority of participants had their first contact with the area during their bachelor’s years (22.8%), but without attending a discipline or engaging in research work. Around 37.6% of participants had their first contact with the field while doing research in their doctoral years, while 22% had it during their master’s years. Around 11.0% studied genomics and/or bioinformatics for the first time during their master’s years, while approximately 8.8% did it during their doctoral studies. One-fourth of the respondents (25.0%) had a scholarship during their bachelor’s, master’s, doctoral degree, and post-doctoral position.

The participants were also asked about their work experience. The majority of them work in the university or another public institution in an area related to genomics and/or bioinformatics (59.0%), and around 10.0% of participants are not working at the moment. Around 8% of the respondents work with genomics and/or bioinformatics in the private sector. Most of them collaborate with other national (51.0%) or international (35.0%) research groups, while a minority has no collaboration (10.0%). 

Around 91% of the participants said to access or generate sequencing data (DNA, RNA, or proteins) in their research, and 28.9% of the participants make use of data that is available in public databases, while only 6.2% hire private services. Furthermore, 87.0% of those believe that the generation of sequencing data changed the area in the last ten years. Most of the participants access bioinformatics tools as a user (36%), while 27.0% generate databases and 11.0% generate bioinformatics programs.

### Participants’ perceptions

As mentioned in section 3.2.2, the open-ended questions of the questionnaire were evaluated by applying content analysis and sentiment analysis techniques. 

### Perceptions about the genomics and/or bioinformatics current situation in Brazil

The first question to be evaluated was:*How do you see the field in the area of genomics and/or bioinformatics in Brazil?* This question aimed to gather participants’ perceptions about the current situation of the field in Brazil, and it resulted in 525 valid responses after data cleaning. Few procedures were used to analyze the data. After performing the stemming processing over the data, the most common stems were identified. The most frequent stem is “grow” (18.3% of the cases), which is found in words such as “growing”, “growth”, and the verb “to grow”. When looking at this word in context, we find positive perceptions about the field in Brazil, such as the following:

*Case 145: A field that is GROWING more and more and is recognized as very relevant to medicine, agriculture, pharmaceuticals, among others.*

In some other cases, the stem “grow” shows up as part of a positive but concerned perception. The following example highlights the potential of the research area’s growth in Brazil, but the participant demonstrates to be worried about the lack of financial investment: 

*Case 57: Brazil has great potential to GROW in the area and could be a great producer and exporter of knowledge and research in the areas of genomics and bioinformatics, but there is a lack of incentives and funding.*

The next most popular stem is “develop” (13.0% of cases), occurring in responses related to the development of the area in Brazil. Some comments highlight aspects relevant to the attraction of professionals to the research area, such as the creation of new courses:

*Case 34: Exponential DEVELOPMENT. The impact is currently occurring at the undergraduate and internships level. More courses are being offered in the earlier training periods. It has attracted professionals from outside Biology, especially Physicists and professionals with computer training.*

Another popular stem is “promis” (10.8% of cases), which occurs as part of words like “promising”. The high frequency of this stem seems to be related to the stem “grow”, as most of the cases state the quick development of the field in Brazil, as illustrated by the following example:

*Case 120: It is one of the most PROMISING fields in science since the number of data increases with each new platform developed, and, in contrast, the number of well-qualified bioinformatics/statistics professionals is still very limited.*

**Figure 2 -:**
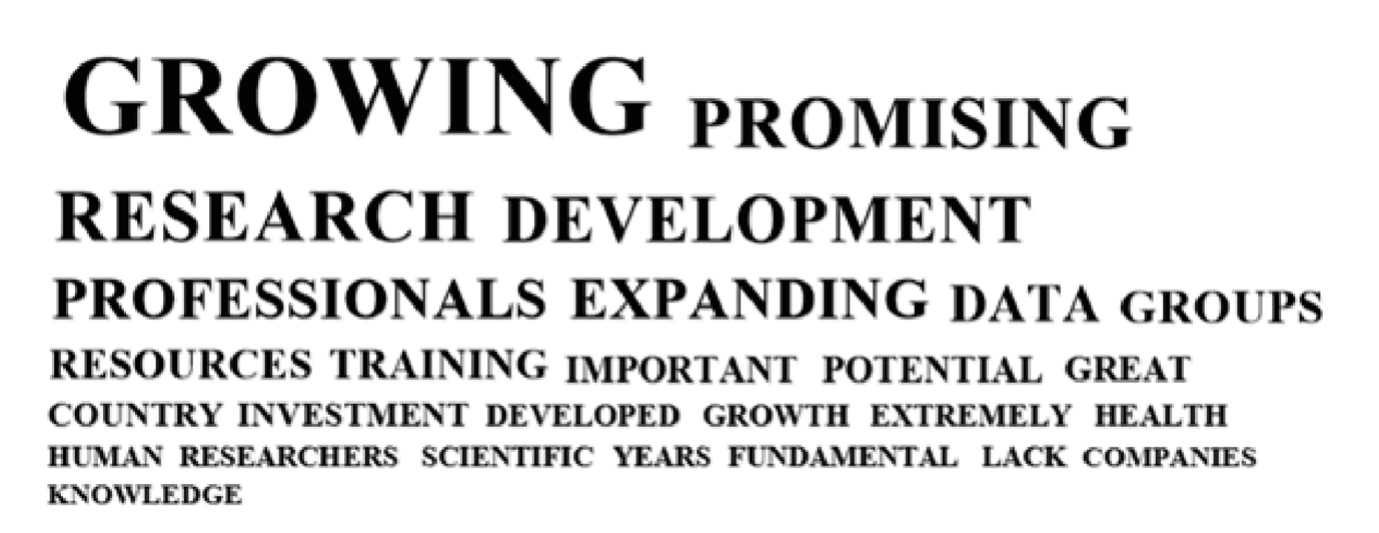
Word cloud illustrating the most frequent words occurring in the participants’ responses referring to the current situation of the field of genomics and/or bioinformatics in Brazil.

The following word cloud illustrates the most frequent whole words, which occur in the analysis at least 15 times (Figure 2).

**Figure 3 -:**
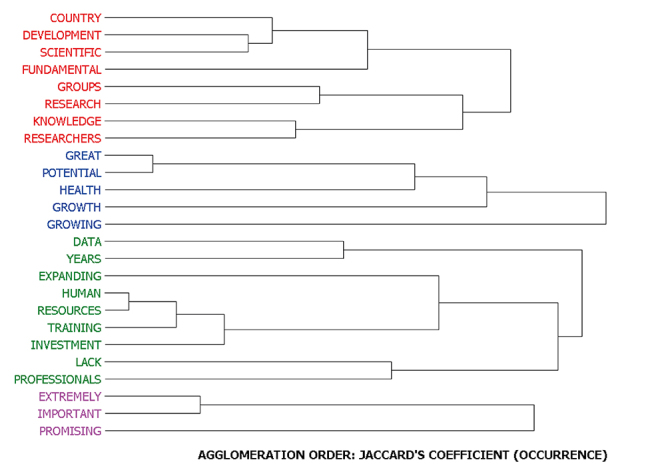
Clusters of words co-occurring in the same sentence part of the responses referring to the current situation of the field in Brazil

The analysis also allowed us to identify clusters of words that co-occurred in the same sentence. A sentence is included in the cluster when containing at least two of the words that are part of that cluster. The following image illustrates the clusters identified (Figure 3).

Table 3 illustrates an example of each cluster. 

**Table 3-:** Examples of sentences present in each cluster identified in the responses about the current situation of the area of genomics and/or bioinformatics in Brazil.

Cluster	Number of Cases	Example
Cluster 1	47	Case 186 - FUNDAMENTAL for SCIENTIFIC-technological DEVELOPMENT of RESEARCH in the COUNTRY.
Cluster 2	15	Case 423 - Today, this is a very promising field and has GREAT GROWTH POTENTIAL with regard to HEALTH and the environment.
Cluster 3	46	Case 480 - There is a LACK of INVESTMENT in computational infrastructure and in the TRAINING of HUMAN RESOURCES (especially for software developers).
Cluster 4	11	Case 379 - EXTREMELY IMPORTANT for the development of new techniques and discoveries.

The answers to the question were also evaluated using sentiment analysis based on the OpLexicon dictionary. The results show that around 47.0% of the comments about the current scenario of the field in Brazil are positive, 38.0% are neutral, and 15.0% are negative. Table 4 illustrates examples of each sentiment classification.

**Table 4 -:** Examples of sentiment analysis classification results performed on the current situation of the field in Brazil.

Sentiment classification	%	Example
Positive	46.0%	Case 398 - Very positive, with the training and qualification of several research groups in different institutions.
Neutral	41.0%	Case 230 - In the current circumstances, we need to have a lot of creativity. There is a huge amount of data being generated by science that is ignored by ‘bench’ scientists. These data could even be explored as training for students and researchers. Many scientific questions can have answers among these data.
Negative	13.0%	Case 541 - When it comes to research, I don’t see much prospect of change. Bioinformatics is still widely seen as a way to “save your work”, when everything else did not work, or to make it more beautiful to publish; the whole question of screening and previous study of a topic for better targeting of research are forgotten. This is a perspective difficult to change and is very much related to living in a moment when there is very little incentive for scientific development, with an even worse scenario when it comes to works that involve genomics/bioinformatics only.

### Perceptions about the genomics and/or bioinformatics future situation in Brazil

The second question to be evaluated was:*What is your vision for the future for genomics and/or bioinformatics in Brazil*. This question aimed to gather participants’ perceptions about the future situation of the field in Brazil, and it resulted in 507 valid responses after data cleaning. The analysis shows that the most common stem is “research” (19.3% of cases). Some researchers highlighted the potential of research in the field, but the need for more investments and funding:

*Case 2: I believe it will improve, but as long as the remuneration and investment in RESEARCH are low, the tendency is to continue to lose recently graduated researchers abroad.*

Others expanded the needs of research funding to other areas as well:

*Case 197: Any vision of the future in the RESEARCH area in Brazil is very obscure and not very encouraging with the current government. I do not see a promising future for science in the country as a whole, including bioinformatics. However, I think that bioinformatics may be less affected than other areas that need funds for data generation and sequencing.*

Furthermore, the second most common stem is “invest” (11.1% of cases), which occurs in words such as “investment” and the verb “to invest”. These stems occur in responses where the need for investment in the field is highlighted: 

*Case 2: I believe it will improve, but as long as the remuneration and INVESTMENT in research are low, the tendency is to continue to lose recently graduated researchers abroad.*

Others suggest a positive view of the future of the area in Brazil considering investments already made:

*Case 39: Extremely promising, with the emergence of new private-sector research and INVESTMENTS groups in the creation of new companies specializing in the area.*

The next most frequent stem is “develop” (10.8% of cases), which occurs in words like “development”. Some responses highlighted the need of developing national technologies:

*Case 7: Stop relying so much on technology and professionals from abroad, and be more consolidated in Brazil, both in the existence of professionals able to execute and DEVELOP new technologies in the area*

Others believe this development is possible if there is enough investment:

*Case 229: If there is support for research, very promising, with international partnerships for the DEVELOPMENT of products and shared databases*

**Figure 4-:**
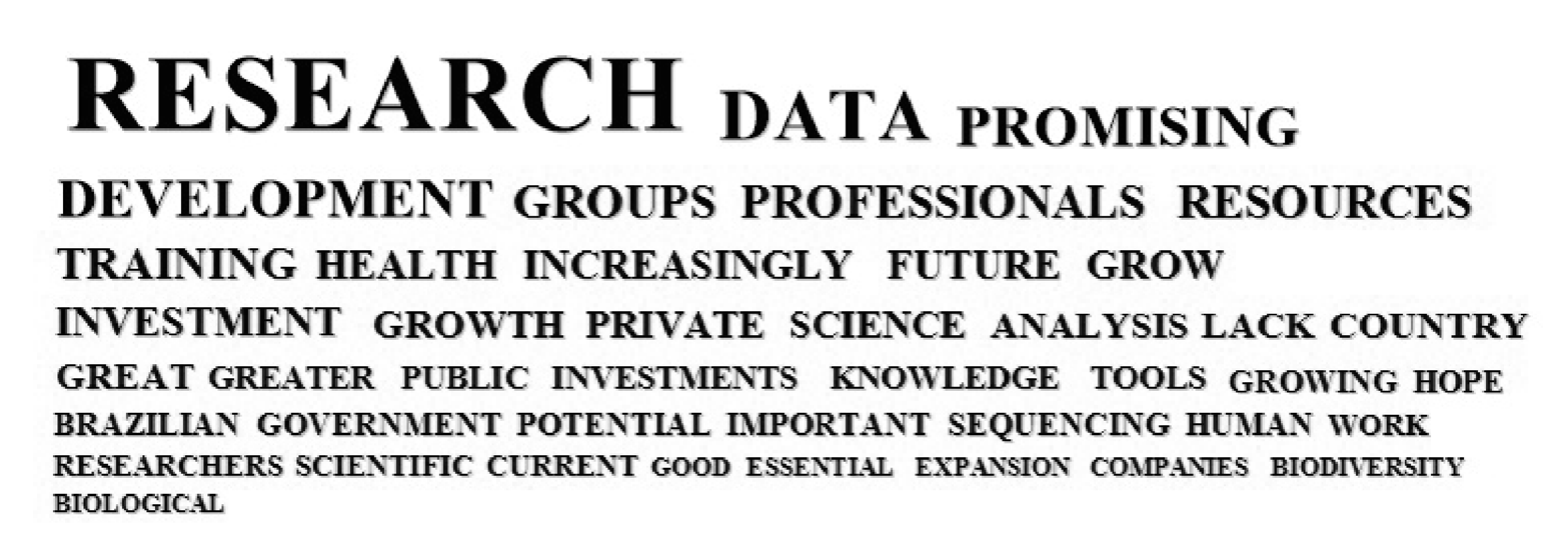
Word cloud illustrating the most frequent words occurring in the participants’ responses referring to the future situation of the field of genomics and/or bioinformatics in Brazil

Figure 4 illustrates the most frequent whole words in the responses about the future of genomics and/or bioinformatics in Brazil.

**Figure 5 -:**
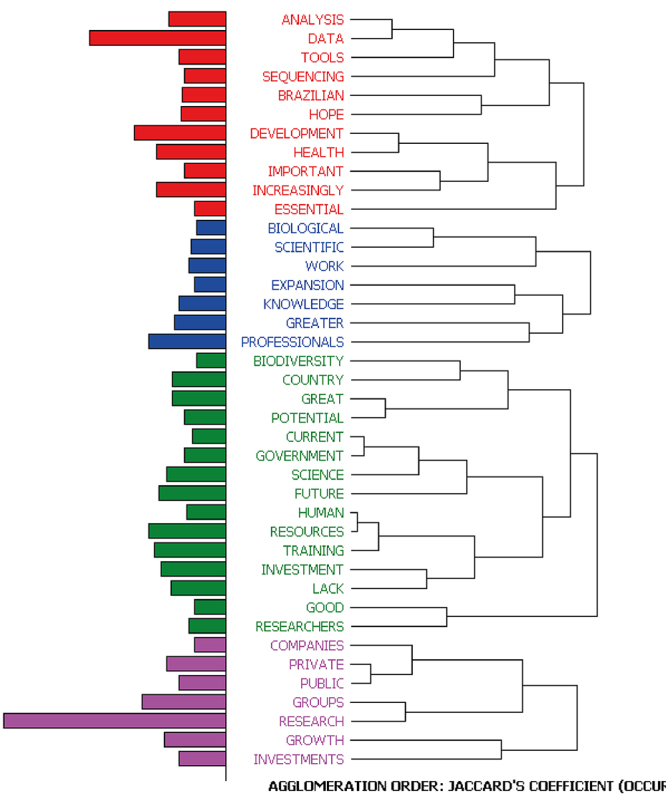
Clusters of words co-occurring in the same sentence part of the responses referring to the future situation of the field in Brazil.

The analysis was also extended to identify clusters with words co-occurring in the same sentence (Figure 5). Table 5 illustrates an example of each cluster.

**Table 5-:** Examples of sentences present in each cluster identified in the responses about the future situation of the area of genomics and/or bioinformatics in Brazil.

Cluster	Number of Cases	Example
Cluster 1	62	Case 23 - I HOPE that we can have more DATA on genetic variation of the BRAZILIAN population and also that we have ANALYSIS TOOLS with a good user experience (I think most of the TOOLS, not only BRAZILIAN ones, are hard to use. Who knows a BRAZILIAN SEQUENCING equipment?
Cluster 2	22	Case 133 - This will bring a GREATER demand for PROFESSIONALS qualified to WORK with this data.
Cluster 3	88	Case 182 - I believe it will improve, but as long as the remuneration and INVESTMENT in research are low, the tendency is to continue to lose recently graduated RESEARCHERS abroad.
Cluster 4	53	Case 39 - Extremely promising, with the emergence of new PRIVATE sector RESEARCH and investment GROUPS in the creation of new COMPANIES specialising in the area.

The sentiment analysis of the responses about the future of genomics and/or bioinformatics in Brazil resulted in around 48.0% of positive comments, 15.0% of negative comments, 37.0% of neutral comments. Table 6 illustrates examples of each sentiment classification. 

**Table 6 -:** Examples of sentiment analysis classification results performed on the future situation of the field in Brazil.

Sentiment classification	%	Example
Positive	46.0%	Case 38 - Extremely promising, with the emergence of new research and investment groups from the private sector in the creation of new companies specialised in the area.
Neutral	41.0%	Case 453 - I believe that all professionals will have to know the minimum bioinformatics for the development of their research in the near future.
Negative	13.0%	Case 107 - Difficult, resources are still limited and more collaboration is lacking, including data availability

### Perceptions about genomics and/or bioinformatics situation in the world

The third question to be evaluated was:*How do you see the field in the area of genomics and/or bioinformatics in the world?*This question aimed to gather participants’ perceptions about the field of genomics and/or bioinformatics outside Brazil, and resulted in 507 valid responses after data cleaning. Once again, stemmed words were used to gather the most common words used by the respondents. The most frequent stem is “grow” (22.3% of the cases). A number of responses focused on talking about the area in the world by comparing the productivity of Brazilian science with other countries’:

*Case 111: As there is a greater investment by private companies (and government, in some places in the world), I believe that the area of genomics/bioinformatics will have a more advanced GROWTH when compared to Brazil. There will be the development of new sequencing platforms and greater “popularisation” of their use. Along with this, I believe that there will also be a greater demand (and also development) for more advanced computational architectures than the current one.*

Others highlighted the progress and growth of the area in emerging countries:

*Case 143: Exponential GROWTH with the great development of tools, new technologies, and applications in the most diverse areas of knowledge. Expansion in Latin America and emerging countries.*

The next most popular stem was “data” (9.5% of cases), mostly focusing on the amount of data being generated by the current analysis. Once again, some participants compared the Brazilian capacity of generating data with the other countries’ capacity: 

*Case 339: More advanced than in Brazil, mainly due to the ease in generating data in countries with cutting-edge science.*

Others shared their experiences in working in other countries. These comments tend to emphasize how advanced the research in genomics and bioinformatics can be in some countries, highlighting the possibilities of development when researchers have easy access to the technologies they need:

*Case 355: Very developed. I had the opportunity to do an internship in a Microbiome laboratory in the Czech Republic that only worked with cutting-edge genomics and bioinformatics. Researchers in the laboratory made it possible long-term work, and therefore, very relevant. In other areas, such as oncology, genomics and bioinformatics are considered the tools for decoding cancer. Large sequencing projects are common in the United States and Europe, with the possibility of free access to at least part of the DATA.*

However, there seems to be a concern in relation to the large amount of data being generated by countries with access to cutting-edge technologies. Some researchers seem to worry about how this data is being analyzed and if these analyses can provide insightful results:

*Case 19: I think it is well developed, but I have read many articles, mainly with sequencing data for bacterial genomes, with which I work, with doubtful or little clarified DATA. You see many articles now with studies on everything, many data being published but few with more in-depth and relevant content.*

Others stated that the lack of proper analysis might be due to the lack of trained researchers, leading to poor data analysis:

*Case 152: Promisingly and expanding. I note that the generation of genomic DATA is no longer a problem and can be done quickly and cheaply abroad, but there is a lack of people to do the data analysis (bioinformatics) with quality.*

**Figure 6 -:**
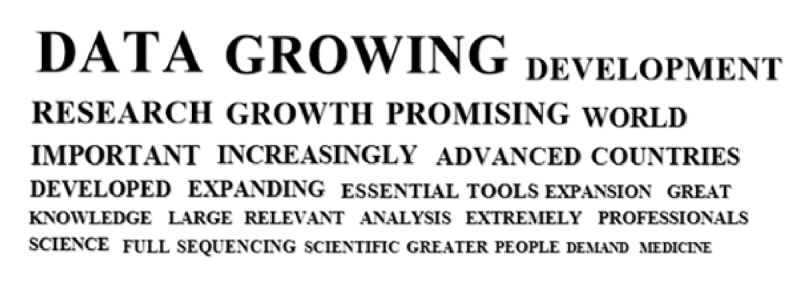
Word cloud illustrating the most frequent words occurring in the participants’ responses referring to the current situation of the field of genomics and/or bioinformatics outside Brazil.

The following word cloud illustrates the most frequent words in the responses about the situation of genomics and/or bioinformatics in the world (Figure 6).

**Figure 7-:**
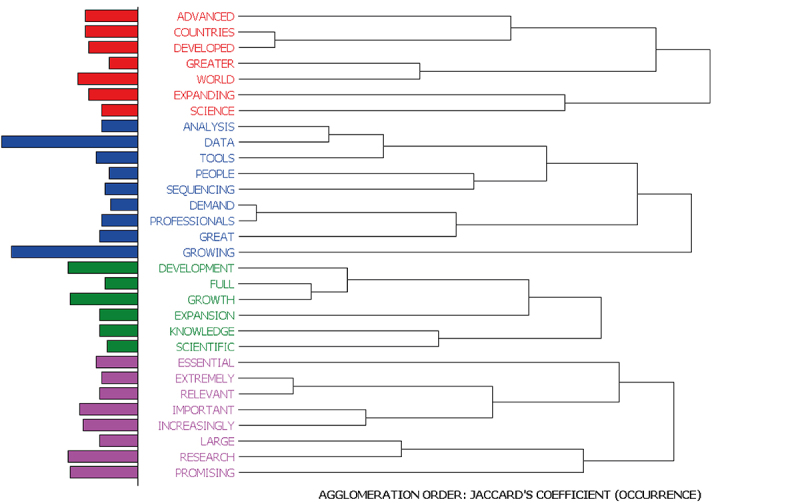
Clusters of words co-occurring in the same sentence part of the responses referring to the situation of the field outside Brazil.

The following shows the cluster of words that co-occurred in the same sentence in the responses about the situation of the field outside Brazil (Figure 7), followed by an example of each cluster in Table 7. 

**Table 7-:** Examples of sentences present in each cluster identified in the responses about the situation of the area of genomics and/or bioinformatics outside Brazil.

Cluster	Number of Cases	Example
Cluster 1	25	Case 383 - The advances in these areas are also much GREATER around the WORLD compared to Brazil.
Cluster 2	50	Case 45 - The available SEQUENCING techniques allow to generate a very large amount of DATA, which directly leads to the need for ANALYSIS by bioinformatics, therefore an area that keeps GROWING.
Cluster 3	26	Case 335 - It is very cheap abroad to do this type of analysis, so the trend is the GROWTH and EXPANSION of data volume for each species, allowing mainly more KNOWLEDGE of functional genomics.
Cluster 4	41	Case 409- LARGE private RESEARCH centers, companies and universities, working together to develop RESEARCH and commercial strategies.

The sentiment analysis of the responses about the situation of genomics and/or bioinformatics outside Brazil resulted in around 48.0% of positive comments, 7.0% of negative comments, 45.0% of neutral comments. Table 8 illustrates examples of each sentiment classification. 

**Table 8-:** Examples of sentiment analysis classification results performed on the situation of the field outside Brazil.

Sentiment classification	%	Example
Positive	47.0%	Case 67 - Well developed, recognised and valued, with numerous groups and important research centres, both nationally and internationally, entirely dedicated to the generation of knowledge, development and application in the area, in addition to training and generation of human resources in scientific computing, both for natives and foreigners.
Neutral	46.0%	Case 148 - Genomics and/or bioinformatics data are abundant. The biggest challenge is in the analysis of these data.
Negative	7.0%	Case 226 - Expanding. However, it is noticeable that each group leader sought/chose their study model. As a result, there is countless fragmented research on the same topic. There is a real need for planning in groups focused on the same theme.

### Perceptions about the milestones of genomics and/or bioinformatics in Brazil

One of the questions present in the questionnaire was related to the main achievements of the field in Brazil: *What are the scientific and technological milestones in the area of genomics and/or bioinformatics that you consider important in Brazil in the last 20 years?*

This question resulted in 474 valid responses after data cleaning, and the responses were evaluated by content analysis. Based on a careful review of the responses, codes were retrieved and used to classify the texts. A number of 32 participants stated that they do not know or remember what the milestones are, and 4 participants said there are no milestones in the area in Brazil. These responses were excluded from the analysis. The most frequent code is related to programs and projects developed in the area (87.0%). Among those, the most cited project (22.3% of cases) was the ONSA network. The next most frequent category is the sequencing of specific organisms carried out by Brazilian researchers, such as human cancer, sugar cane, and other agriculture-related organisms.

Table 9 summarises the results of this content analysis, showing the main milestones of the field in Brazil.

**Table 9 -:** Categories adopted to classify the milestones of genomics and/or bioinformatics in Brazil.

Category	Description	Percentage
Programs and projects	Implementation of programs and projects in the area, such as the Human Genome Project.	87.0%
Sequencing of organisms	Highlights of the main organisms sequenced, such as those relevant to areas like agriculture and health	66.0%
Technology	Development of new technologies, such as sequencers, high-performance computers and servers.	60.0%
Funding	More financial investment on research in the area	4.0%
Education and training	Development of formal education courses (bachelor’s and post-graduation degree) and training programs.	13.0%
Industry and institutions	Creation of new institutions and companies /start-ups in the area	15.0%
Techniques	Development of new sequencing techniques.	10.0%

**Figure 8 -:**
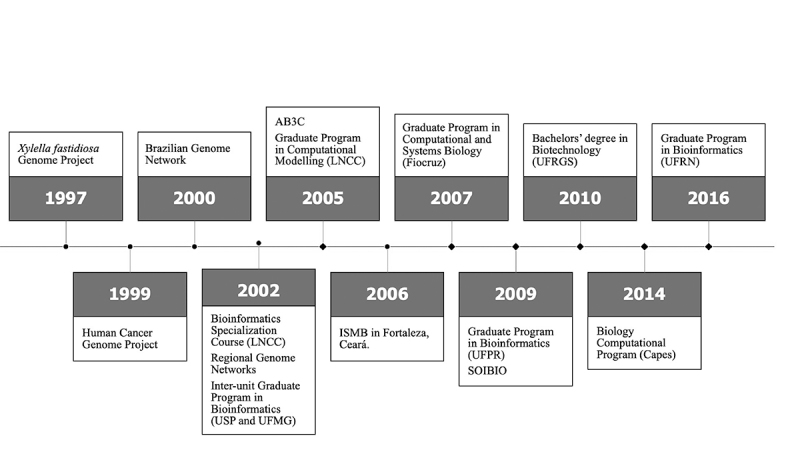
The timeline highlights the main milestones of the development of this research area in the country. The abbreviations stand for AB3C: Brazilian Association of Bioinformatics and Computational Biology, Capes: Coordination for the Improvement of Higher Education Personnel, Fiocruz: Oswald Cruz Foundation, ISMB: Intelligent Systems for Molecular Biology, LNCC: National Laboratory for Scientific Computing, SOIBIO: Iberoamerican Society for Bioinformatics, UFMG: Federal University of Minas Gerais, UFPR: Federal University of Paraná, UFRGS: Federal University of Rio Grande do Sul, UFRN: Federal University of Rio Grande do Norte, USP: University of São Paulo.

The milestones highlighted by the participants of this study are in line with the historical achievements of the genomics and bioinformatics research in Brazil, as illustrated by Figure 8.

After stating what the past milestones of the field of genomics and/or bioinformatics in Brazil are, the participants stated what they wish for the field in the future by answering the question:*If you had a “wish list” of what could be done to improve research in genomics and/or bioinformatics in Brazil, what would you change? For example, sequencing platform, bioinformatics, training, collaborations, financing?*Once again, we performed content analysis classifying the text based on codes retrieved from the responses (Table 10). In line with some of the results identified previously, most of the participants responded they hope to get more financial investments in the field (56.8%), besides a reduction of research input prices and taxes, facilitating their purchase. Furthermore, a significant number of respondents reinforced the need for investments in formal education by creating more courses and specific training in the area of genomics and/or bioinformatics (48.2%).

**Table 10 -:** Categories adopted on the classification of the respondents’ wish lists.

Category	Description	Percentage
Funding	Responses state the need to funding for research and development in the area. It also includes the reduction of the price of research inputs and equipment, besides investment in the industry providing larger teams and start-ups creation.	56.8%
Formal education	Responses related to the need of implementing new bachelor’s, master’s and PhD degrees, besides training for specific technologies.	48.2%
Collaboration	States the wishes for more collaboration between research groups, institutions and private companies/industry.	33.3%
Infra-structure	Comments about the need of new equipment researchers would like to have in their hosting university or company, such as supercomputers or research inputs.	32.5%
Career	A higher number of job positions for researchers and professionals in the area, higher salaries etc.	9.6%
Communication	Better communication about the area to the general public, besides including aspects related to the area to the basic school curriculum.	5.7%

## Discussion

The present study aimed to deliver an overview of the field of genomics and bioinformatics based on the perception of Brazilian experts. The field is relatively new and combines a variety of other areas, such as biology, computer science, statistics and mathematics, as an effort to answer biological queries. When looking at the demographics outcomes of our study, the results suggest there is a balance in terms of participants’ gender. This is a positive result, especially considering that, in the world, only 28.0% of researchers in Science, Technology, Engineering and Mathematics (STEM) are female (UNESCO, 2018). However, few of the respondents are over 50 years old, which might suggest that the field is still young in Brazil. There is a concentration of researchers in São Paulo and very few outside the South and Southeast regions of Brazil. That is in line with other areas of research, especially considering the high concentration of research funding in these areas. According to the GEOCAPES - Georeferenced Information System, the number of research scholarships in the South and Southeast areas of Brazil can be up to 10 times higher than in the North and Northeast areas. A recent report about science production in Brazil shows that 70% of the expenses with research and development are concentrated in São Paulo (Centro de Gestão e Estudos Estratégicos, CGEE, 2021. This can also be attributed to the relevance of FAPESP for the state, considering the foundation provides funding support not only for research and innovation, but also for granting scholarships for graduate students and tools for the insertion of researchers in companies. While setting up any new program, whether academic or service orientated, is a challenging task, together with some established resources that will aid in the development of a bioinformatics program in different Brazilian regions. In an era driven by data science, the need for bioinformatics research and service activities within academic institutes is essential to ensure equal opportunities for competitive research funding. Brazil has attempted to decrease the difference between regions. For example, it is mandatory that at least 30% of all science and technology funding go to the North, Northeast and Midwest regions of the country (Decree-Law 719/69). Among the thirteen bioinformatics networks funded by Capes from 2014 to 2019, one, one and two were from the North, Midwest and Northeast regions, respectively. A direct consequence of this action was the establishment of the Ms/PhD program at the Federal University of Rio Grande do Norte in 2016. Since the importance of genomics and bioinformatics continues to grow, initiatives like the one above are important and should be continued”. 

Most participants have a background in Biological Sciences and tend to have their first contact with the area during their PhD. Since computer scientists and mathematicians are important for the development of the area, the above information is a sign that specific policies for higher involvement of these professionals should be developed. “Using and producing genetic material” was selected by 91.0% of the participants. This illustrates the importance of data access and generation to this field. However, as highlighted by the respondents in the open-ended questions, this large amount of data needs to be adequately evaluated to generate proper insights. The reduction of the costs of biological systems profiling leads the field to a path of “big data”, and the development of efficient methodologies, such as machine learning techniques and computational power, are necessary to generate valuable results (Greene et al., 2014; Yin et al., 2017; Nazipova et al., 2018; Aron et al., 2021). 

The views of the field in Brazil are positive, both for the current situation and for the future. However, compared to the opinions about the field abroad, the respondents had almost double negative opinions. The positive views can be identified by the perceptions of a growing, promising research field. It is clear there is a need for funding investments for the future, as stated in the participants’ wish list. Nevertheless, this scenario seems to be unlikely - the year 2021 was hit by a reduction in science funding (Pires, 2020; Quintans-Júnior et al., 2021). This can be a disadvantage not only for the research field but also for the economy, as the global market for genomics is expected to reach USD 54.4 billion by 2025. Even with financial challenges, Brazilian researchers managed to work and develop relevant programs and projects in the area, which were highlighted by the participants when talking about the milestones in the field. National projects generate important results considering the specificities of the country’s industry and population, allowing researchers to search for personalized solutions (Salzano, 2018; Giugliani et al., 2019). 

Although research output in genomics and bioinformatics has significantly increased in the last decade in Latin America, impact and quality is still a matter of concern. A comparative analysis of the data presented here with similar initiatives in other Latin American countries (Blas et al., 2011; Bicudo, 2016; De Las Rivas et al., 2019; Zambrano-Mila et al., 2019; Armenta-Medina et al., 2020) has revealed some interesting patterns. One of them is the recognition for a better characterization of the genetic structure of the region’s population, a theme that can be deeply explored by genomics and bioinformatics. Another common theme among different countries is the lack of computational infrastructure, which could be minimized by establishing new transnational networks (and an improvement in funding of the existing networks) with common infrastructure. The establishment of undergraduate bioinformatics electives can be implemented within the long-term context of building significant capacity to create a graduate bioinformatics program. Investments in infrastructure support can make important contributions to the advancement of biomedical research, agriculture, among others, through the association of different applications of bioinformatics techniques. Ongoing support through Networking also presents opportunities for collaborative research. This type of initiative proved to be extremely important for the emergence and maintenance of the area in Brazil and should be permanently encouraged. Finally, genomics/bioinformatics communities in several countries recognized the huge potential of both areas and the need for continuous educational efforts. 

The study does not aim to be exhaustive and has limitations. First, we only covered around half of the population expected to be working in the field, so results reflect the perceptions of a sample of the professionals active in the area. Furthermore, there are still limitations related to text mining studies in the Portuguese language, especially due to the limitation of the lexicon available. Nevertheless, this study contributes to the body of knowledge, offering details about the current situation and future expectations of the professionals from the field considering a diverse analysis that includes multiple methods. This type of analysis allows the development of public policy and industry initiatives that can support the development of the field based on the perceptions of their stakeholders. This manuscript and the accompanying data will be forwarded to the major funding agencies in Brazil. 

## Supplementary Material

The following online material is available for this article:

Appendix - Research survey for data collection about experts’ perceptions of genomics and bioinformatics in Brazil.
