# Supplementary material for: The past, present and future of genomics and bioinformatics: A survey of Brazilian scientists
Source: Genet Mol Biol. 2022 Jun 1;45(2):e20210354. doi: 10.1590/1678-4685-GMB-2021-0354 (PMC9169998; doi:10.1590/1678-4685-GMB-2021-0354)
Supplement: Appendix - [file 1415-4757-GMB-45-2-e20210354-s1.pdf]

Genetics and Molecular Biology
https://doi.org/10.1590/1678-4685-GMB-2021-0354

Supplementary Material to "The past, present and future of genomics and bioinformatics: A survey of Brazilian scientists"

Appendix - Research survey for data collection about experts' perceptions of genomics and bioinformatics in Brazil.

Genomics and bioinformatics in Brazil

This research aims to conduct a survey of data on the current scenario, problems, advances and impacts of research in the area of genomics and/or bioinformatics in Brazil. In this study, we would like to get more information about you, the importance of this area in your research, your vision and prospects for the future.

This study is aimed at researchers, students and professionals who are involved in these areas. There are no right or wrong answers. Your collaboration will be important to outline the development of the areas in the country.

Thank you for your participation!!!

Ana Tereza Vasconcelos

Bioinformatics Laboratory – LNCC/MCTI

Luisa Massarani

Coordinator of the National Institute of Public Communication of Science and Technology

Sandro Souza

Brain Institute - Federal University of Rio Grande do Norte

*Required

1. How long have you worked in genomics and/or bioinformatics? *

    - less than 5 years
    - between 5 and 10 years
    - between 10 and 15 years
    - more than 15 years
    - never acted.

2. What is your gender? *

    - Female
    - Male
    - I prefer not to declare
    - Another

3. How old are you? *

4. Where do you live? (City/State) *

5. Thinking about your education from literacy to high school, you would say that THE MOST part of your studies took place: *

    - In a public institution
    - In a private institution
    - Half of the studies were in a public institution and half in a private institution

6. Thinking about your college education from the beginning of undergraduate studies to your last level of study, you would say that THE MOST part of your studies took place: *

- In a public institution
- In a private institution
- Half of the studies were in a public institution and half in a private institution

7. Still thinking about your university education, check in the following table, for all levels of education, if you are attending or have already completed and in case you have already completed what was the year of completion (check all the alternatives that currently correspond to your professional performance).

	Currently studying	Completed up to 5 years ago	Completed between 05 and 10 years ago	Completed more than 10 years ago
Bachelor's degree				
Specialization				
Master's degree				
PhD				
Post-doctoral				

8. Thinking from the beginning of your undergraduate course, at which stage of your education have you had contact with, studied or participated/conducted research on topics related to genomics and/or bioinformatics? (check all alternatives that correspond to your professional performance at that time)

	Had contact without attending a module or participating in a research project in genomics and/or bioinformatics	Studied genomics and/or bioinformatics in some module	Participated/conducted genomics and/or bioinformatics research
Bachelor's degree			
Specialization			
Master's degree			
PhD			
Post-doctoral			
Short course			

9. What area(s) of knowledge is your university education? Mark all areas of knowledge of your education from undergraduate studies to date. *

- o Food Science
- o Agricultural Sciences
- o Veterinary Medicine
- o Zootechnics / Fishing Resources
- o Biodiversity
- o Biological Sciences
- o Physical Education
- o Nursing
- o Pharmacy
- o Medicine
- o Nutrition
- o Dentistry
- o Collective Health
- o Anthropology / Archaeology
- o Political Science and International Relations
- o Sciences of Religion and Theology
- o Education
- o Philosophy
- o Geography
- o History
- o Psychology

- o Sociology
- o Public and Business Administration, Accounting and Tourism
- o Architecture, Urbanism and Design
- o Communication and Information
- o Law
- o Economy
- o Urban and Regional Planning / Demography
- o Social Service
- o Arts
- o Linguistics and Literature
- o Astronomy / Physics
- o Computer Science
- o Geosciences
- o Mathematics / Probability and Statistics
- o Chemistry
- o Engineering
- o Biotechnology
- o Environmental Sciences
- o Teaching
- o Interdisciplinary
- o Materials

Other:

10. Thinking about the different stages of your third level education, from the beginning of your undergraduate studies until now, you had a scholarship or research funding: (check all the alternatives that correspond to your professional performance at that time) *

- o During undergraduate studies
- o In the specialization
- o During the Master's degree
- o In the doctorate
- o During the Postdoctoral
- o Does not apply

11. Currently you: (check all alternatives that correspond to your professional performance at this time) *

- o Works in a private company in an area related to genomics and/or bioinformatics.

- Works in a private company in an area not related to genomics and/or bioinformatics.
- Works in the foundation, university or public research body in an area related to genomics and/or bioinformatics.
- Works in a foundation, university or public research body in an area not related to genomics and/or bioinformatics.
- None of the above alternatives
- Not currently working
- Other:

12. And currently you: *

- Study or research some area related to genomics and/or bioinformatics in a private company
- Study or research some area related to genomics and/or bioinformatics at a foundation, university or public research body.
- Does not study or research areas related to genomics and/or bioinformatics.
- Does not study or research but is interested in working in a field related to genomics and/or bioinformatics.

13. For you when did the genomics and/or bioinformatics area started in Brazil? *

- before 2000
- between 2000 and 2010
- after 2010

14. Do you access or generate sequence data (DNA/RNA or proteins) in your search? *

- Yes
- No

15. If your answer was “yes” to the question above: Has the generation of sequence data changed your research in the last decade? *

- o Increased
- o There was no change

16. To analyze your data, you use: platform/program/generated data (check all alternatives that correspond to your professional performance): *

- o available in national public institutions
- o available in international public institutions
- o developed by your group
- o available under hiring a private service

17. Considering your research, you think genomics and/or bioinformatics is: *

- o extremely relevant
- o relevant
- o a little relevant
- o I use it occasionally

18. In which area do you apply your knowledge in genomics and/or bioinformatics? (check all alternatives that correspond to your professional performance) *

- o Health
- o Agrarian
- o Biotechnology

- Environmental
- I don't apply it
- Other:

19. Regarding your knowledge in genomics and/or bioinformatics as you define yourself: (check all alternatives that correspond to your professional performance) *

    - Experience in bench data generation
    - Experience in data processing for bioinformatics analysis
    - Experience as a user of bioinformatics tools
    - Experience as a developer of bioinformatics programs

20. In your search you: (check all alternatives that correspond to your professional performance) *

    - collaborates with other research groups in the field of genomics and/or bioinformatics in Brazil
    - collaborates with other research groups in the field of genomics and/or bioinformatics abroad
    - has no collaboration with other genomics and/or bioinformatics research groups

21. How do you see the field in the area of genomics and/or bioinformatics in Brazil? *

22. What are the scientific and technological milestones in genomics and/or bioinformatics that you consider important in Brazil in the last 20 years? *

23. What is your vision of the future for genomics and/or bioinformatics in Brazil? *

24. How do you see the field in the field of genomics and/or bioinformatics in the world? *

25. If you had a "wish list" of what could be done to improve research in genomics and/or bioinformatics in Brazil, what would you change? For example, sequencing platform, bioinformatics, training, collaborations, funding? *

26. If you wish, please post additional comments here.

27. Name (optional):

28. E-mail (optional):
